# Paediatric Orbital Fractures: The Importance of Regular Thorough Eye Assessment and Appropriate Referral

**DOI:** 10.1155/2013/376564

**Published:** 2013-11-18

**Authors:** Karim Kassam, Ishrat Rahim, Caroline Mills

**Affiliations:** Department of Oral & Maxillofacial Surgery, Northwick Park Hospital, London HA1 3UJ, UK

## Abstract

The paediatric orbital fracture should always raise alarm bells to all clinicians working in an emergency department. A delay or failure in diagnosis and appropriate referral can result in rapidly developing and profound complications. We present a boy of childhood age who sustained trauma to his eye during a bicycle injury. Acceptance of the referral was based on no eye signs; however, on examination in our unit the eye had reduction in visual acuity, no pupillary reaction, and ophthalmoplegia. CT scan suggested bone impinging on the globe and the child was rushed to theatre for removal of the bony fragment. Postoperatively no improvement was noted and a diagnosis of traumatic optic neuropathy was made. An overview of factors complicating paediatric orbital injuries, their associated “red flags”, and appropriate referral are discussed in this short paper.

## 1. Introduction

Orbital fractures in children can present the attending clinician with a diagnostic dilemma. This stems from suboptimal patient cooperation and differing physiological response to trauma especially orbital. It is important that the clinician anticipates these differences when assessing a child and makes the correct diagnosis so as not to compromise the outcome.

## 2. Case Presentation

A boy of childhood age was referred from an A&E department to a maxillofacial unit following a fall from a bicycle. The right side of his face had fallen into the handlebar of the bicycle before his head hit the ground. The patient was transferred to the accepting hospital some five hours after the initial incident following a period of neurological observation. The referral was accepted by the maxillofacial team on the basis of information given by the referring clinician: that the patient had been cleared of head and C-spine injury, that there were no eye signs on departure, and that a CT scan had been performed. Glasgow Coma Scale was said to be 15/15.

**Figure 1:**
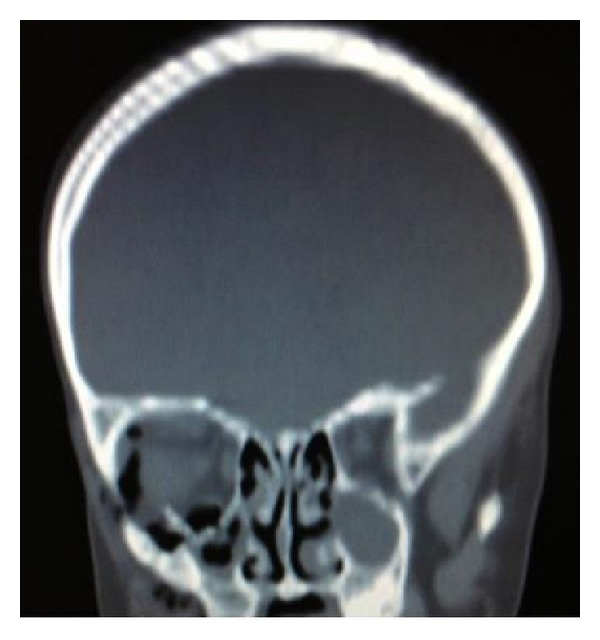
Coronal slice showing impingement of floor into globe.

**Figure 2:**
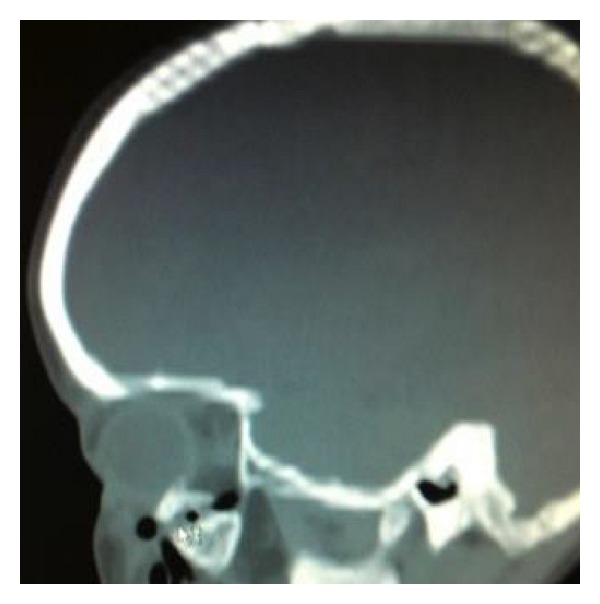
Sagittal Slice showing impingement of floor into posterior globe.

**Figure 3:**
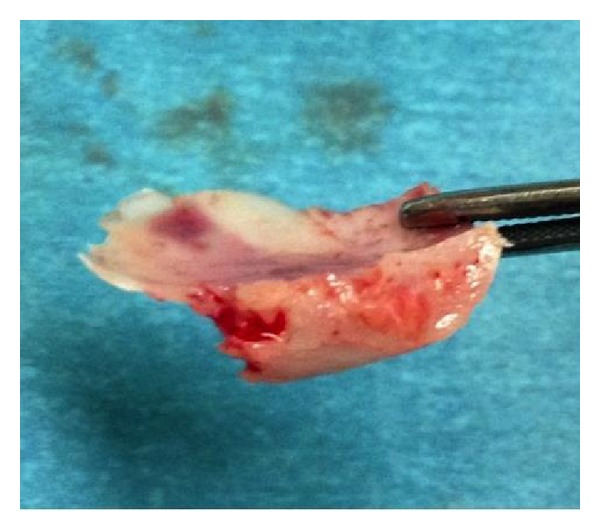
Intraoperative picture illustrating bone removed (anterior part is part of infraorbital rim).

On examination by the maxillofacial team at the accepting hospital to which he was transferred, forcefully separating the lids of the affected eye was necessary to facilitate full assessment. The affected eye had a fixed and dilated pupil, with the patient complaining he was unable to see. On review of the CT head, it was clear that a fractured bony fragment was impinging directly onto the globe (Figures 1 and 2). There had been no mention of the relation of this fragment to the globe in the CT report from the referring hospital. The patient was rushed to theatre by the maxillofacial team for removal of the bony fragment (Figure 3) and then transferred urgently to a hospital with ophthalmology onsite. On further examination of the referring clinician's clinical notes, visual acuity was not documented and it was clear from the neurology observations that the pupil of the right eye had not been assessed for at least two hours prior to transfer, the reasons being cited as “unable” and the eye being “closed.”

After a specialist ophthalmologist input, the diagnosis was traumatic optic neuropathy. In immediate and short-term followup, there was no improvement in visual acuity.

## 3. Discussion

Paediatric orbital fractures are complicated a number of factors.

- More flexible, elastic bone: this often results in greenstick fractures or buckling injuries of the bone. The ends of a bony fracture in a child frequently recoil back into position thereby entrapping adjacent extraocular muscle or soft tissue [1, 2].
- Pronounced oculocardiac reflex: this is a phenomenon which is seen frequently in fractures of the orbital floor and is often marked in children. The reflex is mediated by connections between the sensory afferent fibres of the ophthalmic division of the trigeminal nerve and the visceral motor nucleus of vagus nerve [1]. This results in bradycardia and hypotension, accompanied by headache, nausea, and vomiting. The effects of this response can often be confusing for the emergency doctor and misdiagnosed as head injury.
- Suboptimal patient cooperation: the understandably suboptimal cooperation of the injured child can make assessment of the affected eye difficult. Young children are not able to describe their vision particularly well or explain their visual symptoms.

We describe three main “red flags” that clinicians should have in the back of their mind when assessing a child with an orbital injury. 

*(1) The Orbital “White-Eye” Blowout Fracture.* The medial wall and floor of the orbit are the most frequent locations of fracture, as in adults. As mentioned above, in children, this often results in entrapment of the adjacent extraocular muscle. Classic signs of this type of fracture are diplopia and limitation on a gaze, of which the direction is dependent upon the extraocular muscle(s) trapped. The “white-eye” phenomenon refers to the lack of clinical evidence of soft tissue trauma, such as swelling, ecchymosis, or subcon- junctival haemorrhage. Bradycardia, hypotension, headache, nausea, and vomiting are all culminations of the previously mentioned oculocardiac reflex that is more pronounced in children. Early surgical intervention is required in order to prevent necrosis of any entrapped muscles and subsequent visual impairment. 

*(2) Impingement of Fractured Bone on The Globe*. Although the globe's position within the orbit protects it from injury in many situations, the impact and direction of certain traumatic injuries can result in bony fragments becoming impinged on or impacted in it. Needless to say, this direct trauma to the globe, particularly to its posterior segment, is associated with a very high frequency of permanent visual loss, due to globe rupture [3]. The patient is likely to experience pain on any movement of the eye, and decreasing visual acuity and light perception are likely to develop rapidly. 

*(3) Retrobulbar Haemorrhage*. It is a rare emergency which most commonly arises from orbital trauma but can also be a complication of zygomatic complex fractures and orbital surgery. Essentially, as the name suggests, bleeding. 

Prompt diagnosis and management are essential in preventing loss of vision. Immediate surgical intervention will entail lateral canthotomy and inferior cantholysis under local or general anaesthetic [4, 5]. Orbital decompression and evacuation of haematoma will form the definitive surgical management. Medical management can be commenced immediately using intravenous mannitol, dexamethasone or methylprednisolone, and acetazolamide [4]. Knowledge of medical management is paramount for emergency medicine doctors to “buy” time while waiting for specialty senior support.

History should include the mechanism, direction, and velocity of injury as these are important in assessing the likelihood of direct trauma to the globe and the severity of fracture. Needless to say, the priority in examination is thorough assessment of the eye. Physical examination should be performed in a systematic manner to avoid missed injury in accordance with advanced trauma life support protocol.

Specifically in terms of maxillofacial assessment, one should examine the head, neck, scalp, face, and periorbital tissues to assess for lacerations, oedema, foreign bodies, or sensory deficits, such as infraorbital paraesthesia in the setting of orbital blowout fractures [6]. Tenderness to palpation around the orbital rim and any step deformities should be noted as well.

Every effort should be made to examine the eyes fully; this may mean having to pry the affected eye open to overcome the inevitable swelling and bruising. It must also be remembered that each eye should be checked separately using a physical partition in the centre-line to ensure accuracy of assessment and that excessive pressure on the globe should be avoided.  Table 1 below illustrates a recommended outline by the author for examination.

In extreme cases where forceful opening of the eye is not possible, at the very least, the consensual light reflex should be assessed by shining a light into the affected eye and checking for pupillary constriction in the contralateral eye. This test will suffice in testing the optic nerve of the affected eye; as a bright light can still pass through a closed lid and elicit a pupillary response. If the pupil of the contralateral eye is not responsive, first rule out a nerve palsy in this eye by checking its direct light response. If this can be excluded, damage to optic nerve in the affected eye is highly likely.

Computed tomography of the orbits is the imaging modality of choice and should be requested urgently. If a CT of the entire head is required due to suspected head injury, 1 mm coronal slices should be included in the request so that any orbital fracture can be assessed simultaneously and optimally. If there are any concerning features of the orbital fracture, it is the duty of the radiologist reviewing and reporting the scan to raise alarm to the referring team. Needless to say, the referring team should also review any imaging themselves; reliance on the report alone is unsatisfactory. Urgent referral to ophthalmology should be made if any vision-threatening features are discovered, for example, foreign body impaction or impingement of bony fragments on the globe.

In the ideal situation, every hospital would have both an ophthalmology and a Maxillofacial team on-call and onsite. However, this is frequently not the case and transfer of the patient to another hospital is often required. It is of paramount importance that the doctors and nurses in the emergency department perform regular eye observations whilst awaiting transfer; as this has the propensity to change the required management entirely. A maxillofacial team may well have accepted a referral based on initial assessment but it must be remembered that visual acuity and pupillary response are dynamic and can deteriorate rapidly. If any eye signs are detected whilst awaiting transfer, an urgent ophthalmology review will take precedence over any maxillofacial intervention.

In this particular case the diagnosis made by ophthalmology was traumatic optic neuropathy (TON). Typically, the initial clinical ophthalmic findings include decreased visual acuity (VA) or blindness and a relative afferent pupillary defect (RAPD) which our patient developed. This occurs when there is disruption around the optic canal resulting in either compression of the optic nerve, shearing forces to the nerve as it passes through the canal, or haematoma formation within the nerve itself. Untreated, it can render the patient blind and the diagnosis needs to be made early to allow the best chance of visual recovery. Generally the prognosis in terms of visual recovery is poor; however, there are no well-designed studies illustrating whether surgical decompression or steroids compared to observation alone is the ideal treatment. A recent Cochrane review established no clear scientific basis for the use of steroids in traumatic optic neuropathy [7]. Ford et al. reported that the incidence of TON in the UK is similar in adults (1.01 per million) and children (0.99 cases per million), with the majority of cases occurring after relatively minor head injuries. Male preponderance, sports injuries, and falls were the most frequent causes [8]. Lee et al. concluded that there was little evidence to suggest benefit of steroid treatment for isolated optic nerve injury, where visual loss is immediate. They advocated that surgical treatment should be directed by the radiological findings. Orbital haematomas causing optic nerve compression should be acutely evacuated, and where there is a bony fragment impinging on the nerve from an optic canal fracture, then endoscopic optic nerve decompression should be considered and similarly optic nerve fenestration in the case of intrasheath haemorrhage [9].

In our case there was obvious impingement of bone on the globe where it was felt that removal of the bony fragment would be in the patient's best interest as well as steroid treatment. However, studies have shown that traumatic optic neuropathy with concomitant orbital fractures tend to have more severe visual loss [7, 10].

The outcome of the patient may have been the same following the immediate injury but the importance of repeated accurate assessment cannot be overemphasised as well as appropriate and timely referral.

## 4. Main Learning Points

1. All paediatric orbital fractures require urgent attention due to the obvious proximity of the globe and the potential implications on vision. Of particular importance are the orbital “white eye” blowout fracture, impingement of bony fragments on the globe, and retrobulbar haemorrhage.
2. CT of the head or orbits is the imaging modality of choice, which should include 1 mm coronal slices and be reported urgently. (This should not delay emergency surgical management to save vision.)
3. Appropriate referral is absolutely essential for the correct management of paediatric orbital fractures.
  - Regular and repeated assessment of the eye during the patient's stay in A&E is of critical importance. It must be remembered that visual acuity and pupillary response are dynamic and can rapidly deteriorate. If at any stage, any eye signs are detected clinically, an urgent ophthalmology review will always take precedence over any maxillofacial intervention.
  - Any vision threatening abnormalities discovered on CT imaging should be red-flagged to the requesting clinicians so that urgent action can be taken. Again, ophthalmologic intervention in the first instance will be essential in order to maximize functional outcome.

## Figures and Tables

**Table 1:** Ophthalmological examination.

	Findings requiring urgent ophthalmological review	How to assess in the paediatric patient
Visual acuity	Reduced/loss of vision	(i) Paediatric Snellen chart (ii) Counting examiner's displayed fingers (iii) Detecting hand motion (iv) Ask patient if they can see

Pupillary light response: direct and consensual	Sluggish/loss of direct reflex Sluggish/loss of consensual reflex	As in adults

Swinging light test	Presence of rapid afferent pupillary defect/Marcus Gunn pupil in affected eye	As in adults

Pupil size	Dilatation	As in adults

Pain	Pain	(i) Direct questioning

Fields	Restriction Diplopia Pain on movement	(i) Use an object of interest (ii) Ask patient about double vision and pain

Colour	Desaturation/loss of red reflex	Not possible in the very young (i) Direct questioning (ii) Matching of coloured objects

Position of globe	Proptosis Enophthalmos	As in adults
